# Interplay of the IGFBP-3 polymorphism and serum levels of IGF-1/IGFBP-3 with hormone receptor subtypes in patients with breast cancer among Palestinian women

**DOI:** 10.1371/journal.pone.0350553

**Published:** 2026-06-01

**Authors:** Heba Mohammed Arafat, Tengku Ahmad Damitri Al-Astani Tengku Din, Noorazliyana Shafii, Rosediani Muhamad, Ihab Naser, Nahed Al Laham, Ohood Mohammed Shamallakh

**Affiliations:** 1 Department of Chemical Pathology, School of Medical Sciences, Health Campus, Universiti Sains Malaysia, Kubang Kerian, Kelantan, Malaysia; 2 Breast Cancer Awareness and Research Unit, Hospital Pakar Universiti Sains Malaysia, Health Kampus, Kubang Kerian, Kelantan, Malaysia; 3 Department of Family Medicine, School of Medical Sciences, Health Campus, Universiti Sains Malaysia, Kubang Kerian, Kelantan, Malaysia; 4 Department of Clinical Nutrition, Faculty of Applied Medical Sciences, Al-Azhar University- Gaza, Gaza City, Palestine; 5 Department of Laboratory Medicine, Al Azhar University-Gaza, Gaza City, Palestine; 6 Department of Medical Laboratory Sciences, Faculty of Health Sciences, Islamic University of Gaza, Gaza City, Palestine; Medical University of South Carolina, UNITED STATES OF AMERICA

## Abstract

Breast cancer remains a global public health challenge. This study aimed to investigate the relationships between serum levels of insulin-like growth factor binding protein 3 (IGFBP-3), insulin-like growth factor 1 (IGF-1), the IGFBP-3 A-202C polymorphism and hormone receptor subtypes: estrogen receptor (ER), progesterone receptor (PR), and human epidermal growth factor receptor 2 (HER-2) with respect to breast cancer risk in Palestinian women. A cross-sectional study was conducted on 112 newly diagnosed, histopathologically confirmed breast cancer patients. Data collection included structured interviews and laboratory analyses, including biochemical, genetic, and immunohistochemical assessments. The tumor tissue samples were analyzed for ER, PR, and HER-2 status. SPSS version 28 was used for all data analysis. A high prevalence of hormone receptor positivity was observed. Among the breast cancer patients, 87.5% were ER positive and 84.8% were PR positive. A total of 75.9% were HER-2 negative. The IGFBP-3 A-202C genotype is significantly associated with PR and combined ER/PR positivity (*p* = 0.020). Patients with ER(+)/PR(+) status had significantly higher serum IGF-1 and IGFBP-3 levels (*p* ≤ 0.001), with IGF-1 levels positively correlated with hormone receptor status (*r*_*s*_ = 0.232, *p* ≤ 0.001) and advanced disease stages, moderately with Stage III (*r*_*s*_ = 0.191, *p* ≤ 0.001) and weakly correlated with Stage IV (*r*_*s*_ = 0.119, *p* = 0.029). These findings suggest that IGF-1 and IGFBP-3 may have potential as candidate biomarkers for breast cancer risk and progression. Integrating genetic, biochemical, and hormone receptor status provides novel insights into breast cancer biology and may support future research on personalized prevention and treatment strategies, particularly in resource-limited settings such as the Gaza Strip.

## 1. Introduction

According to GLOBOCAN 2022, female breast cancer was the second most frequently diagnosed cancer worldwide, accounting for approximately 11.6% of all cancer cases, with an estimated 2.3 million new cases and 670,000 deaths globally [1,2]. While breast cancer affects women worldwide, its burden is particularly concerning in low- and middle-income countries, where early detection and access to treatment are often limited [3]. One such region is the Gaza Strip—a densely populated area facing ongoing socioeconomic and political challenges—where breast cancer has emerged as a significant and growing public health concern. Recent data revealed that 394 new breast cancer cases were registered, accounting for 19.2% of all newly diagnosed cancers. Among women, breast cancer is the most commonly diagnosed cancer, accounting for 36.9% of all female cancer cases [4] and is the leading cause of cancer-related death in the region [5]. Owing to delayed diagnosis and limited access to specialized care, many women present with advanced-stage disease, contributing to higher mortality rates and poorer outcomes [6].

Understanding the biological and molecular characteristics of breast cancer is essential for improving patient management, particularly in resource-constrained settings such as Gaza [7]. Among the critical factors influencing breast cancer development and progression are hormonal and growth-regulatory pathways [8]. The insulin-like growth factor (IGF) axis, comprising IGF-1 and its principal binding protein, IGFBP-3, plays a significant role in cell proliferation, differentiation, apoptosis, and tumorigenesis [9]. IGF-1 exerts mitogenic and anti-apoptotic effects [10], whereas IGFBP-3 modulates IGF-1 bioavailability and possesses independent growth-inhibitory effects [9]. Dysregulation of this axis has been implicated in several malignancies, including breast cancer [11].

In addition to serum biomarker levels, genetic variations within the IGF axis have attracted considerable attention for their potential role in modifying breast cancer susceptibility [12]. The IGFBP-3 A-202C (rs2854744) polymorphism, located in the promoter region of the IGFBP-3 gene, has been shown to influence transcriptional activity and circulating protein levels [13]. Studies have explored the association between the IGFBP-3 A-202C polymorphism and breast cancer risk [14–16]; however, the results remain inconsistent and appear to vary by population and genetic background.

Hormone receptor status, including ER, PR, and HER-2, is a cornerstone of breast cancer classification and plays a critical role in guiding treatment decisions and predicting prognosis [17]. Tumors that are positive for ER and PR generally respond better to endocrine therapy and are often associated with more favorable outcomes [18]. In contrast, triple-negative breast cancer (ER-, PR-, HER-2-) tends to show more aggressive behavior and is associated with limited treatment options [19]. Accordingly, accurate assessment of these markers is essential for optimal clinical management of breast cancer. Immunohistochemistry (IHC) is the preferred method for evaluating their expression [20], as it is relatively cost-effective, widely accessible, and provides both predictive and therapeutic information [21]. Emerging evidence suggests that components of the IGF axis may be associated with hormone receptor status. For example, estrogens regulate the expression of IGF-1 and the IGF-1 receptor [22], whereas IGFBP-3 may exert either synergistic or antagonistic effects depending on the tumor context [23,24].

Given the biological relevance of the IGF axis in breast cancer progression and the clinical significance of hormone receptor status, understanding the interplay among serum IGF-1 and IGFBP-3 levels, IGFBP-3 A-202C genotypes, and hormone receptor subtypes may provide valuable insights into breast cancer pathogenesis and help identify potential biomarkers for risk stratification and personalized treatment. However, such investigations remain scarce in the Palestinian population, particularly in the Gaza Strip, where environmental, nutritional, and genetic factors may uniquely influence disease development.

Therefore, this study aimed to address this knowledge gap by examining the relationship among serum IGF-1 and IGFBP-3 levels, the IGFBP-3 A-202C polymorphism, and hormone receptor subtypes (ER, PR, and HER-2), as well as the interactions among these biomarkers in breast cancer patients from the Gaza Strip. By integrating molecular, genetic, and clinical data, this research seeks to enhance understanding of breast cancer in this underserved population and provide insights that may inform future diagnostic and therapeutic strategies.

## 2. Methods and materials

### 2.1. Study design and participants

**Fig 1:**
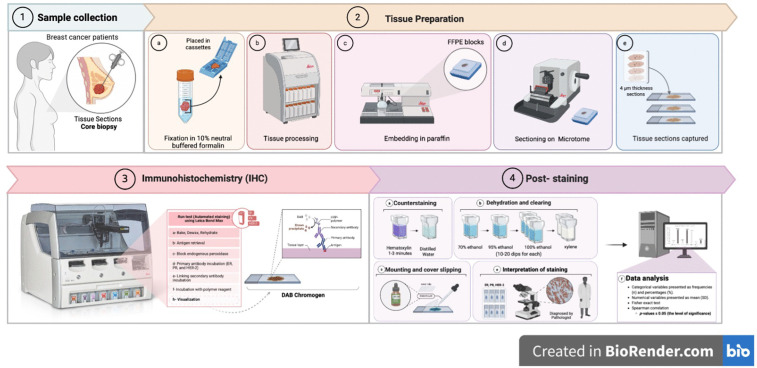
Overview of Methodological‌‌ Workflow https://app.biorender.com/illustrations/66ba80cb0924959867ece918?slideId=3119eec3-8ce4-495c-9207-772779b5471c.

This observational cross-sectional study was conducted from February 3, 2020, to February 2, 2022. The study period encompassed ethical approval, participant recruitment, sample collection, laboratory work, and data analysis. Fig 1 presents an overview of the methodological workflow of the study.‌‌

The study included Palestinian women with newly diagnosed, histopathologically confirmed breast cancer who were identified through computerized medical records at the Oncology Departments of Al-Shifa Hospital and the Turkish Palestinian Friendship Hospital. Eligible participants were treatment-naive at the time of enrollment and had not yet received hormone therapy, chemotherapy, targeted therapy, radiotherapy, or alternative therapy.

This study was conducted using the same cohort as our earlier published work on the IGFBP-3 A-202C genetic variant and serum biomarkers in Palestinian women with breast cancer [25]. In the present analysis, genetic, biochemical, and immunohistochemical data were evaluated to examine the relationship between IGFBP-3 polymorphism, serum IGF-1/IGFBP-3 levels, and hormone receptor subtypes (ER, PR, and HER-2). By integrating genetic, biochemical, and immunohistochemical data, this study provides novel insights into the interplay between molecular biomarkers and hormone receptor status—an aspect not examined in the previous publication. This integrated analysis contributes to a more comprehensive understanding of breast cancer biology and its potential clinical implications in prognosis and treatment stratification.

### 2.2. Ethical approval

Ethical approval was obtained from the Helsinki Committee in the Gaza Strip (PHRC/HC/699/20; approved February 3, 2020). and the USM Human Research Ethics Committee (USM/JEPeM/20020122; approved July 22, 2020), with an extension granted on January 13, 2022. A facilitation letter was also issued by the Palestinian Ministry of Health on October 7, 2020.

Written informed consent was obtained from all participants before their recruitment into the study. To ensure participant privacy and confidentiality, interviews were conducted in a private room, separated from clinical areas, by trained research staff. The participants were informed that their participation was voluntary and that their responses would be kept strictly confidential. All the data collected were anonymized by assigning a unique study identifier to each participant, and all identifying information was removed before data analysis. Data were stored securely on password-protected systems and accessed only by authorized research personnel. Blood samples were collected once per participant and were destroyed after completion of the study.

### 2.3. Data collection, study tool and sampling

Data collection was performed through structured interviews and laboratory analyses, including biochemical, genetic, and immunohistochemical methods. A structured questionnaire was developed to meet the needs of breast cancer patients and was administered during a face-to-face interview.

The sample size was calculated using G*Power software (version 3.1.9.6) based on Correlation: Bivariate normal model. The calculation was performed using a two-tailed test, an expected correlation under the alternative hypothesis (ρH1) of 0.372, a null hypothesis correlation (ρH0) of 0, a significance level (α) of 0.01, and a statistical power (1 − β) of 0.90. Based on these assumptions, the minimum required sample size was 100 participants. To account for a potential nonresponse rate of 10%, the target sample size was increased to 110 participants. A total of 112 patients were ultimately enrolled in the study, ensuring adequate statistical power for the analyses performed.

### 2.4. Blood samples, biochemical analysis, and genetic analysis

Blood samples were collected from participants at Al-Shifa Hospital and Turkish Palestinian Friendship Hospital under standardized sterile conditions and transported to the Palestinian Medical Relief Society for biochemical analysis.

A total of five milliliters (mL) of whole blood was collected into plain tubes, centrifuged for ten minutes at 3,000 rpm, and serum was separated. Serum levels of IGF-1 and IGFBP-3 were measured via analytical kits on the MAGLUMI 800 series fully autochemiluminescence immunoassay analyzer (Snibe, China), according to the manufacturer’s instructions. Results were reported in ng/ml for IGF-1 and µg/ml for IGFBP-3. All samples were assayed in single runs.

For DNA extraction and subsequent single-nucleotide polymorphism (SNP) genotyping, 3 mL of whole blood samples were collected in ethylenediaminetetraacetic acid (EDTA) tubes and stored at −20°C until DNA extraction for up to one year. Storage at −20°C is commonly used for preserving DNA integrity for PCR-based applications [26]. According to the manufacturer’s recommendations, Genomic DNA was extracted from the blood via the Wizard® Genomic DNA Purification Kit (Promega, USA). SNP genotyping of the IGFBP-3 gene was performed via polymerase chain reaction and restriction fragment length polymorphism (PCR-RFLP) technique. The target region was amplified via previously published primers [27]: forward 5′-CCACGAGGTACACACGAATG-3′ and reverse 5′-AGCCGCAGTGCTCGCATCTGG-3′, generating a 459 bp product.

PCR reactions were performed in a total volume of 25 microliters (µl). Amplification was carried out using a Bio-Rad thermal cycler (USA Biometra) under the following conditions: initial denaturation at 95°C for 5 minutes, followed by 37 cycles of denaturation at 95°C for 30 seconds, annealing at 60°C for 30 seconds, and extension at 72°C for 30 seconds, with a final extension at 72°C for 5 minutes.

PCR products were digested using Alw21I restriction enzyme (Thermo Fisher Scientific, USA) and separated on 2% agarose gel electrophoresis, stained with ethidium bromide, and visualized under ultraviolet (UV) illumination using a gel documentation system (Bio-Imaging Systems, Israel). Fragment sizes were determined using a 100 base pair (bp) DNA ladder (Bioline, UK). Genotypes were assigned based on predefined fragment patterns corresponding to each allele: AA genotype (242 and 162 bp), CC genotype (288 and 162 bp), and AC genotype (288, 242, and 162 bp). The presence of additional non-polymorphic Alw21I restriction sites within the 459 bp PCR product served as an internal control to confirm successful enzyme digestion. To minimize subjectivity and ensure reproducibility, gel images were independently reviewed, and genotype assignments were confirmed based on consistent banding patterns.

### 2.5. Immunohistochemistry (IHC)

Immunohistochemical analysis was performed to evaluate the expression of ER, PR, and HER-2 in formalin-fixed paraffin-embedded (FFPE) breast carcinoma tissue samples. The Leica BOND-MAX fully automated IHC and in situ hybridization staining system (Leica Biosystems, Germany) was used, enabling standardized and reproducible staining through automated processing of up to 30 slides simultaneously, ensuring minimal manual variability and high-quality staining output.

### 2.6. Protocol for automated IHC

A. **Sample processing and sectioning**

Breast carcinoma tissue samples were processed by the histopathology staff at Al-Shifa Hospital and the Turkish Palestinian Friendship Hospital. Tissues were fixed in 10% neutral buffered formalin, dehydrated through graded Ethanol (50% to 100%), cleared in xylene, and embedded in paraffin to produce FFPE blocks.

The FFPE blocks were cooled on a −20°C cold plate and sectioned via Leica RM2235 manual microtome. Sections of 4 µm thickness were floated in a 40–45°C water bath and mounted on silanized charged glass slides, labeled with relevant identifiers and stored at 4°C until staining.

B. **Automated Immunohistochemical (IHC) Staining**

Slides were placed in the BOND-MAX system, where automated deparaffinization was performed at 60°C for 20 minutes to remove paraffin wax and allow effective staining. Staining was carried out via preprogrammed protocols on the BOND-MAX system. The process included several Key steps: (a) Dewaxing, BOND Dewax Solution was used to remove paraffin thoroughly; (b) Rehydration, through graded alcohols to restore tissue moisture; (c) Epitope retrieval, by heat-induced antigen unmasking to reverse formalin-induced cross-links; (d) Blocking, with hydrogen peroxide to inhibit endogenous peroxidase activity; (e) primary antibody incubation, in which specific antibodies against ER, PR, and HER-2 (Table 1); (f) secondary antibody linking, followed by (g) incubation with the polymer reagent and horseradish peroxidase-conjugated polymers; and finally (h) visualization with 3,3’-diaminobenzidine (DAB). A brown precipitate was produced at the antigen sites for microscopic detection.

**Table 1:** Details of the primary antibodies used in this study.

Antibody	Clone/Positive tissue control	Antigen retrieval buffer	Antibody dilution	Supplier	Pre-treatment
ER	Monoclonal antibody/ Endometrium	Epitope Retrieval Solution (pH 6).	1:50	Leica, Germany	Heat
PR	Monoclonal antibody/ Breast carcinoma and endometrium.	Epitope Retrieval Solution 2 (pH 8.9–9.1)	Not required		
HER-2	Monoclonal antibody/ Human BC cell lines	Epitope Retrieval Solution 1 (pH 5.9–6.1)	Not required

C. **Poststaining**

Following staining, the BOND-MAX system was used for automated washing steps using buffer solutions to remove unbound antibodies and excess reagents. Subsequently, counterstaining was carried out manually using hematoxylin, which stains cell nuclei blue. Slides were immersed in hematoxylin solution for 1–3 minutes, followed by thorough rinsing with distilled water. This was followed by dehydration and clearing**,** after which the tissue sections were passed through graded alcohols (70% to 100%) and cleared in xylene to remove moisture and prepare for mounting. Finally, mounting and cover slipping were carried out via Histomount™ medium and glass coverslips, with slides left to dry horizontally at room temperature overnight for proper adhesion.

D. **Interpretation and quantitative assessment of staining**

Experienced pathologists at the Histopathology Departments of Al-Shifa and Turkish Palestinian Friendship Hospitals independently evaluated ER, PR, and HER-2 expression using a light microscope at 20 × magnification. Evaluation was performed using standardized scoring systems. The pathologists were blinded to the participants’ clinical and laboratory data to minimize observer bias. In cases of discrepancy, the slides were jointly reviewed to reach a consensus, ensuring consistency and reliability of scoring.

Quantitative assessment was performed using standardized semi-quantitative scoring systems. The ER and PR expression were evaluated on the basis of the Allred scoring system (Table 2), whereas HER-2 expression was assessed according to the ASCO/CAP guidelines (Table 3). All quantitative results were derived from the evaluation of the full cohort and are reported as frequencies and percentages.

**Table 2:** The results for ER/ PR were interpreted according to the Allred scoring method.

Proportion of score	Observation values	Intensity score given	Final observation
0	None	0	None
1	1%	1	Weak
2	1-10%	2	Intermediate
3	10-33%	3	Strong
4	33-66%		
5	66-100%		
**Total score**		**Interpretation**	
0-2		Negative	
3-8		Positive	

**Table 3:** The results for HER-2 were interpreted according to the ASCO/CAP guidelines.

0	No staining or faint perceptible membrane staining in <10% of tumor cells – Negative for HER-2 receptor.
1+	Incomplete and faint perceptible membrane staining in >10% of tumor cells – Negative for HER-2 receptor.
2+	Weak/moderate complete membrane staining in >10% of tumor cells – Equivocal result and reported as negative
3+	Strong complete membrane staining in >10% of tumor cells – Positive for HER-2 receptors.

E. **Tissue controls**

Each staining run included both positive and negative controls. Positive control tissues known to express ER, PR, or HER-2 were used to ensure proper tissue preparation and staining accuracy. Negative controls were prepared by omitting the primary antibody to confirm staining specificity. Failure of control staining rendered the corresponding test results invalid.

### 2.7. Statistical analysis

IBM SPSS version 28 was used for all the statistical analyses. Descriptive statistics were calculated, with categorical variables presented as frequencies (n) and percentages (%), and continuous variables presented as means ± standard deviation (SD).

Prior to inferential analysis, the distribution of continuous variables was assessed using the Shapiro–Wilk test and visual inspection of histograms and Q–Q plots. Variables that did not meet the assumptions of normality were considered non-normally distributed. Associations between IGFBP-3 genotype and categorical clinicopathological variables were assessed using Fisher’s exact test. Comparisons of serum IGF-1 and IGFBP-3 levels between two groups were performed using the independent-samples t-test, whereas comparisons across more than two groups were performed using one-way analysis of variance, as appropriate. Because several study variables, including hormone receptor status and breast cancer stage, were ordinal or categorical in nature, Spearman’s rank correlation was performed to evaluate correlation between the serum levels of IGF-1 and IGFBP-3, and the clinicopathological features, including ER, PR, and HER-2 statuses, and the stages of breast cancer among patients.

Data were checked for completeness, consistency, and entry errors prior to analysis. Potential outliers were identified by visual inspection of boxplots and verified against the original source records. No biologically plausible confirmed values were excluded from the analysis. Missing values, if present, were minimal and were handled using a complete-case analysis approach. A p value of ≤ 0.05 was considered statistically significant.

## 3. Results

### 3.1. Clinicopathological features of breast cancer patients

Table 4 shows all the clinicopathological features of the breast cancer patients in this study. Among 112 breast cancer patients, 52.7% had right-sided lesions, 44.6% had left-sided lesions, and 2.7% had bilateral lesions. Most patients were diagnosed at stage II (50.9%), followed by stage III (41.1%). The most common presenting symptom was a breast mass (89.3%), followed by pain (28.6%), tingling (22.3%), and changes in breast size or shape (21.4%). Fewer patients reported axillary masses (17.9%), redness (9.8%), retracted nipple (6.3%), or nipple discharge (5.4%). Only 1.8% were asymptomatic upon the discovery of cancer by chance or by regular mammography check.

**Table 4:** Clinicopathological features of breast cancer patients.

Feature			Cases
			*N* = 112
			*n* (%)
**Site of breast lesion**	Left		50 (44.6)
	Right		59 (52.7)
	Both		3 (2.7)
**Stage of breast cancer**	Stage I		6 (5.4)
	Stage II		57 (50.9)
	Stage III		46 (41.1)
	Stage IV		3 (2.7)
**Symptoms of breast cancer**	Breast mass	No	12 (10.7)
		Yes	100 (89.3)
	Mass under axilla	No	92 (82.1)
		Yes	20 (17.9)
	Pain	No	80 (71.4)
		Yes	32 (28.6)
	Tingling	No	87 (77.7)
		Yes	25 (22.3)
	Nipple discharge	No	106 (94.6)
		Yes	6 (5.4)
	Retracted nipple	No	105 (93.8)
		Yes	7 (6.3)
	Redness	No	101 (90.2)
		Yes	11 (9.8)
	Two breasts are not equal in size or shape	No	88 (79.6)
		Yes	24 (21.4)
	Asymptomatic	No	110 (98.2)
		Yes	2 (1.8)

Note. *n* = Frequency;

Data are presented as frequencies (percentages).

### 3.2. Frequency of ER, PR, and HER-2 positivity among women with breast cancer

**Fig 2:**
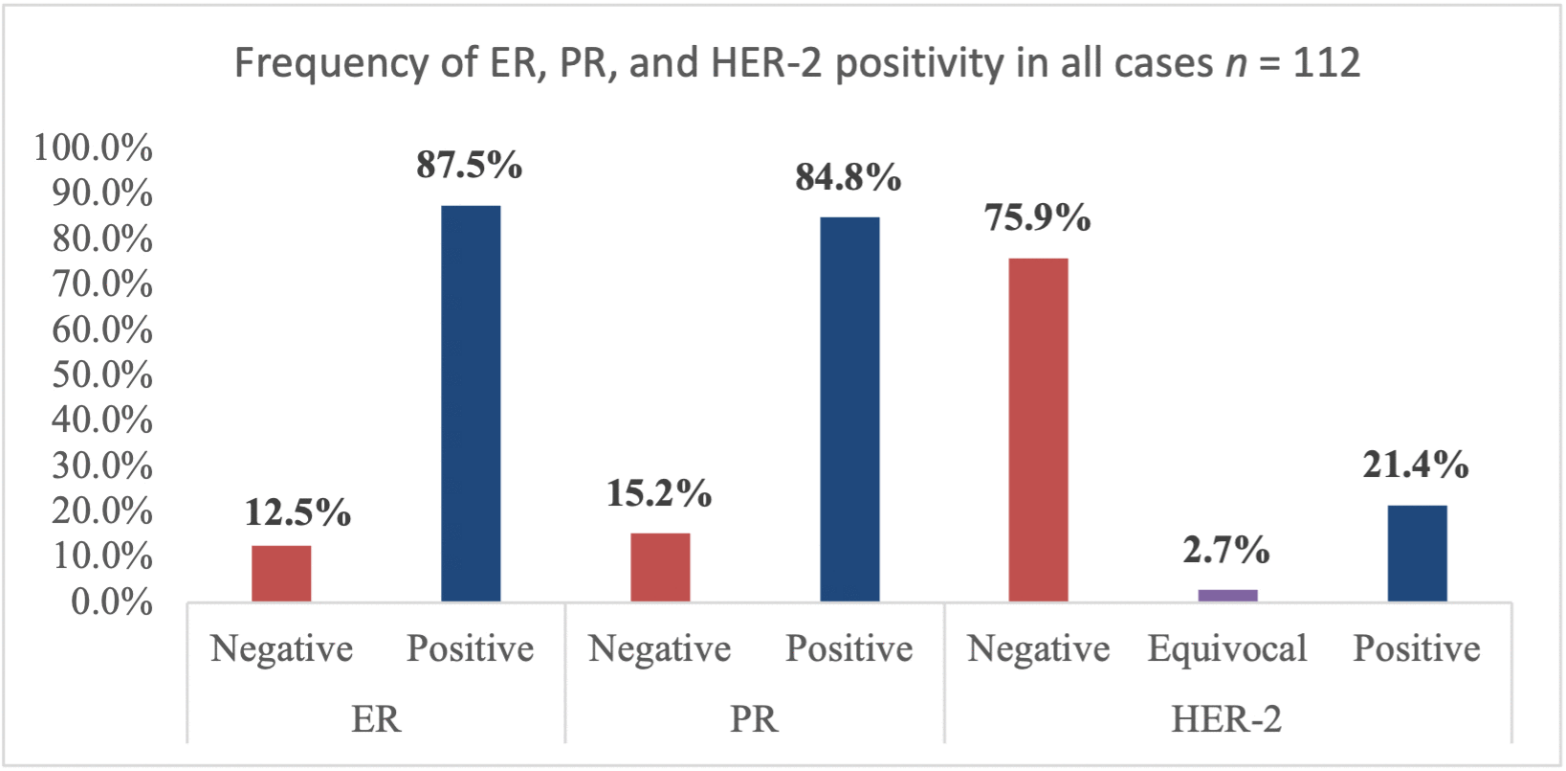
Frequency of ER, PR, and HER-2 positivity. Note. ER = Estrogen receptor; PR = Progesterone receptor; HER-2 = Human epidermal growth factor receptor-2.

**Fig 3:**
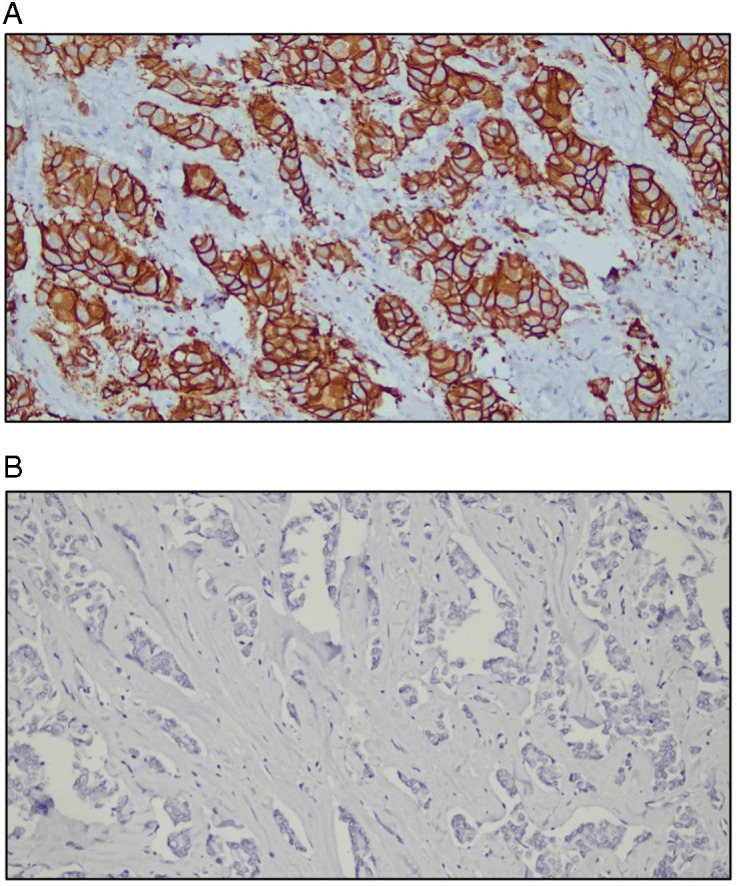
Representative images of HER-2 immunostaining. All the slides were analyzed at 200x magnification, and the scale was 50 mm. Fig 3A: The arrow indicates HER-2-positive staining. Fig 3B: The arrow indicates HER-2-negative staining.

**Fig 4:**
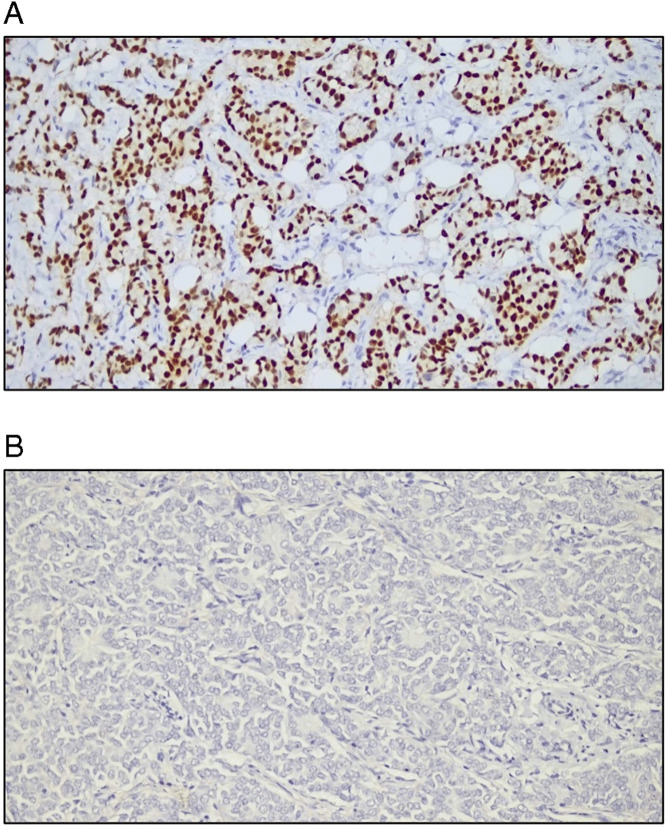
Representative images of ER immunostaining. All the slides were analyzed at 200x magnification and the scale was 50 mm. Fig 4A: The arrow indicates ER-positive staining. Fig 4B: The arrow indicates ER-negative staining.

**Fig 5:**
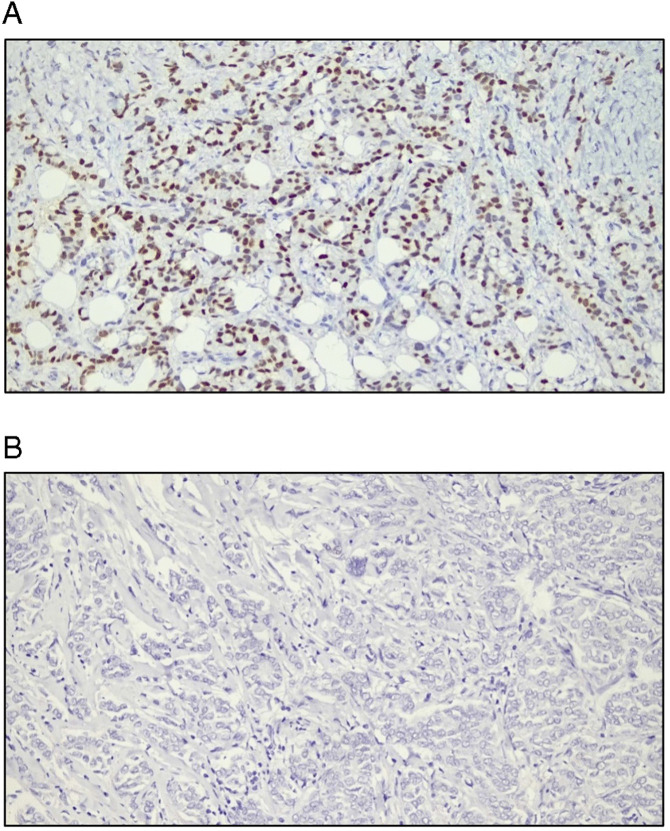
Representative images of PR immunostaining. All the slides were analyzed in 200x magnification and the scale was 50 mm. Fig 5A: The arrow indicates PR-positive staining. Fig 5B: The arrow indicates PR-negative staining.

The IHC results revealed that 87.5% of the patients were ER positive, and 84.8% were PR positive, and 75.9% were HER-2 negative (Fig 2). Combined hormone receptor status reported that 84.8% were ER(+)/PR(+), whereas only 2.7% were ER(+)/PR(-). A total of 12.5% were negative for both. None of the cases were identified as ER(-)/PR(+) (Table 5). Representative immunohistochemical staining patterns for HER-2, ER, and PR expression are shown in Figs 3, 4, and 5, respectively.

**Table 5:** Frequency of combined ER, PR, and HER-2 among women with breast cancer.

Receptors		*n* (%)
**Combined hormone receptor sensitivity**	**ER(+)/PR(+)**	95 (84.8)
	**ER(+)/PR(-)**	3 (2.7)
	**ER(-)/PR(+)**	0 (0.0)
	**ER(-)/PR(-)**	14 (12.5)

Note. ER = Estrogen receptor; PR = Progesterone receptor; HER-2 = Human epidermal growth factor receptor-2; *n* = Frequency.

Data are presented as frequencies (percentages).

### 3.3. Relationships between the IGFBP-3 A-202C genotype and ER, PR, and HER-2 status, and the stage of breast cancer among patients

The relationship between the IGFBP-3 A-202C genotype and breast cancer by considering the pathological features of the cancer, the analysis was performed as shown in Table 6. A significant association was found between the IGFBP-3 A-202C genotype and PR positivity (*p* = 0.020), with higher PR positivity in CC (88.6%) and AC (86.4%) genotypes compared with AA (54.5%). A similar trend was noted for the combined ER(+)/PR(+) status (*p* = 0.020). No significant associations were observed for ER, HER-2 status or cancer stage *(p* > 0.05).

**Table 6:** Relationships between the IGFBP-3 A-202C genotype and ER, PR, and HER-2 status and between the IGFBP-3 A-202C genotype and the stage of breast cancer among patients.

Variables		IGFBP-3 A-202C genotype			Fisher’s exact	*p* value
		Homozygous CC	Heterozygous AC	Homozygous AA		
		*n* (%)	*n* (%)	*n* (%)		
**ER**	Negative	8 (10.1)	2 (9.1)	4 (36.4)	5.199	0.067
	Positive	71 (89.9)	20 (90.9)	7 (63.6)		
**PR**	Negative	9 (11.4)	3 (13.6)	5 (45.5)	7.095	0.020
	Positive	70 (88.6)	19 (86.4)	6 (54.5)		
**Combined hormone receptor sensitivity**	ER(+)/PR(+)	70 (88.6)	19 (86.4)	6 (54.5)	7.095	0.020
	Other	9 (11.4)	3 (13.6)	5 (45.5)		
	ER(+)/PR(-)	1 (1.3)	1 (4.5)	1 (9.1)	3.435	0.207
	Other	78 (98.7)	21 (95.5)	10 (90.9)		
	ER(-)/PR(+)	0 (0.0)	0 (0.0)	0 (0.0)	–	–
	Other	79 (100.0)	22 (100.0)	11(100.0)		
	ER(-)/PR(-)	8 (10.1)	2 (9.1)	4 (36.4)	5.199	0.067
	Other	71 (89.9)	20 (90.9)	7 (63.6)		
**HER-2**	Negative	61 (77.2)	17 (77.3)	7 (63.6)	8.410	0.052
	Equivocal	0 (0.0)	2 (9.1)	1 (9.1)		
	Positive	18 (22.8)	3 (13.6)	3 (27.3)		
**Stage of breast cancer**	Stage I	5 (6.3)	0 (0.0)	1 (9.1)	3.108	0.775
	Stage II	39 (49.4)	13 (59.1)	5 (45.5)		
	Stage III	33 (41.8)	8 (36.4)	5 (45.5)		
	Stage IV	2 (2.5)	1 (4.5)	0 (0.0)		

*Significant at the level of 0.05.

Note. ER = Estrogen receptor; PR = Progesterone receptor; HER-2 = Human epidermal growth factor receptor-2; *n* = Frequency.

Fisher’s exact test.

### 3.4. Comparison of serum IGF-1 and IGFBP-3 with ER, PR, and HER-2 status, and the stages of breast cancer among patients

Comparison between the pathological features of breast cancer and the serum IGF-1 and IGFBP-3 are explored in Tables 7 and 8. There were no significant differences in serum IGF-1 and IGFBP-3 levels when analyzed separately with ER, PR, HER-2 status, or cancer stage (*p* > 0.05). However, significantly higher levels of both IGF-1 (123.78 ± 49.57) and IGFBP-3 (3.61 ± 1.24) were detected in the ER(+)/PR(+) group (*p* ≤ 0.001).

**Table 7:** Comparisons of the serum levels of IGF-1 and IGFBP-3 with those of ER, PR, and combined hormone receptor sensitivity among patients.

Variables		Serum IGF-1 (ng/ml)				Serum IGFBP-3 (µg/ml)			
		Mean ± SD	Mean differences (95%CI)	t statistic (df)	*p*-value	Mean ± SD	Mean differences (95%CI)	t statistic (df)	*p* value
**ER**	Negative	117.99 ± 60.36	−4.23 (−37.87, 29.40)	−0.249 (110)	0.804	4.32 ± 2.11	0.73 (−0.50, 1.97)	1.265 (1.265)	0.226
	Positive	122.22 ± 59.27				3.59 ± 1.23			
**PR**	Negative	112.80 ± 56.81	−10.48 (−41.69, 20.72)	−0.671 (110)	0.504	4.09 ± 1.98	0.48 (−0.56, 1.52)	0.964 (18.307)	0.348
	Positive	120.78 ± 59.69				3.61 ± 1.24			
**Combined hormone receptor sensitivity**	ER(+)/PR(+)	123.78 ± 49.57	−29.50 (−43.16, −15.83)	−4.266 (148.163)	≤ 0.001	3.61 ± 1.24	−0.16 (−0.49, 0.17)	−0.951 (332)	≤ 0.001
	Other	93,78 ± 49.57				3.45 ± 1.45			
	ER(+)/PR(-)	88.60 ± 32.72	13.70 (−48.24, 75.64)	0.435 (332)	0.664	3.00 ± 0.47	0.50 (−1.09, 2.10)	0.617 (332)	0.538
	Other	102.30 ± 54.39				3.50 ± 1.40			
	ER(-)/PR(+)	0				0			
	Other	102.17 ± 54.22				3.49 ± 1.40			
	ER(-)/PR(-)	117.99 ± 60.36	−16.50 (−45.62, 12.60)	−1.11 (332)	0.266	4.32 ± 2.11	−0.86 (−2.09, 0.36)	−1.517 (13.468)	0.152
	Other	101.48 ± 53.94				3.46 ± 1.35			

*Significant at the level of 0.05.

An independent sample t test was applied.

Normality assumption is fulfilled.

Note. SD = standard deviation; ER = estrogen receptor; PR = progesterone receptor; IGF-1 = insulin-like growth factor-1; IGFBP-3 = insulin-like growth factor binding protein-3; df = degree of freedom.

The Data are presented as the means ± SDs.

**Table 8:** Comparison of the serum levels of IGF-1, IGFBP-3, and HER-2 with the stage of breast cancer among patients.

Variables		Serum IGF-1 (ng/ml)			Serum IGFBP-3 (µg/ml)		
		Mean ± SD	F test (df)	*p v*alue	Mean ± SD	F test (df)	*p* value
**HER-2**	Negative	119.95 ± 59.08	0.805 (2)	0.450	3.60 ± 1.37	1.029 (2)	0.361
	Equivocal	90.33 ± 51.14			3.27 ± 1.34		
	Positive	131.78 ± 60.53			4.02 ± 1.40		
**Stage of breast cancer**	Stage I	146.18 ± 60.73	2.385 (3)	0.073	3.51 ± 0.65	0.101 (3)	0.959
	Stage II	109.00 ± 55.16			3.64 ± 1.54		
	Stage III	130.98 ± 62.09			3.73 ± 1.28		
	Stage IV	171.33 ± 30.87			3.94 ± 0.80		

*Significant at the level of 0.05.

One Way ANOVA was applied.

Normality assumption is fulfilled.

Note. CI = confidence interval; HER-2 = human epidermal growth factor receptor-2; IGF-1 = insulin-like growth factor 1; IGFBP-3 = insulin-like growth factor binding protein-3; SD = standard deviation; df = degree of freedom.

The data are presented as the means ± SDs.

### 3.5. Correlation between the serum levels of IGF-1, or IGFBP-3, and ER, PR, and HER-2, and the stage of breast cancer among patients

Correlation between IGF-1 and IGFBP-3 serum levels and patients’ clinicopathological characteristics are shown in Table 9. No statistically significant correlations were observed between IGF-1 serum levels and individual receptor statuses, including ER, PR, and HER-2 (*p* > 0.05 for all). However, a statistically significant positive correlation was found between the IGF-1 level and the combined hormone receptor-positive group ER(+)/PR(+) (*r*_*s*_ = 0.232, *p* ≤ 0.001). This finding suggests that patients with dual hormone receptor positivity may be associated with elevated IGF-1 levels. Moreover, IGF-1 levels were significantly correlated with more advanced disease. Specifically, IGF-1 levels were moderately positively correlated with Stage III breast cancer (*r*_*s*_ = 0.191, *p* ≤ 0.001) and showed a weaker but statistically significant correlation with Stage IV breast cancer (*r*_*s*_ = 0.119, *p* = 0.029). These findings indicate a potential association between higher IGF-1 levels and disease progression. In contrast, the serum IGFBP-3 levels were not significantly correlated with any clinicopathological features (*p* > 0.05).

**Table 9:** Correlations between serum levels of IGF-1 and IGFBP-3 and between ER, PR, and HER-2 status and the stage of breast cancer among patients.

Clinicopathological features		Serum IGF-1 (ng/ml)		Serum IGFBP-3 (µg/ml)	
		Corr. Coeff.	*p* value	Corr. Coeff.	*p* value
**ER**	Negative	−0.017	0.861	0.096	0.314
	Positive	0.017	0.861	−0.096	0.314
**PR**	Negative	−0.061	0.522	0.041	0.666
	Positive	0.061	0.522	−0.041	0.666
**HER-2**	Negative	−0.050	0.603	−0.123	0.195
	Equivocal	−0.093	0.328	−0.036	0.707
	Positive	0.089	0.353	0.143	0.133
**Combined hormone receptor sensitivity**	Other	−0.232**	≤ 0.001	0.036	0.708
	ER(+)/PR(+)	0.232**	≤ 0.001	−0.036	0.708
	Other	0.018	0.744	0.105	0.270
	ER(+)/PR(-)	−0.018	0.744	−0.105	0.270
	Other	–	–	–	–
	ER(-)/PR(+)	–	–	–	–
	Other	−0.055	0.318	−0.090	0.344
	ER(-)/PR(-)	0.055	0.318	0.090	0.344
**Stage of breast cancer**	Stage I	0.102	0.062	0.009	0.877
	Stage II	0.062	0.262	0.043	0.433
	Stage III	0.191**	≤ 0.001	0.079	0.151
	Stage IV	0.119*	0.029	0.051	0.357

*Significant at the level of 0.05.

Note. ER = Estrogen receptor; PR = Progesterone receptor; HER-2 = Human epidermal growth factor receptor-2; IGF-1 = Insulin-like growth factor-1; IGFBP-3 = Insulin-like growth factor binding protein-3; Corr. Coeff. = correlation coefficient; *r*_*s*_ = Spearman correlation

## 4. Discussion

Breast cancer remains one of the most common cancers and a leading cause of cancer-related death among women worldwide [28]. The IGF axis, particularly IGF-1 and IGFBP-3, has been implicated in breast cancer development because of its role in regulating cell proliferation, survival, and apoptosis [29]. In addition, numerous SNPs have been associated with an increased risk of breast cancer, highlighting their potential relevance as molecular biomarkers [30]. Assessment of ER, PR, and HER-2 status also remains a standard component of breast cancer diagnosis, prognosis, and treatment planning [20,31]. In this context, this study set out to determine the associations of the genetic polymorphisms with serum IGF-1 and IGFBP-3 with hormone receptor (ER, PR, and HER-2) status and to understand the interactions between these biomarkers in breast cancer patients.

A prominent finding of this study was the predominance of ER- and PR-positive tumors together with HER-2 negativity in this cohort. This pattern suggests that hormonally responsive tumor subtypes are common, which is clinically relevant because hormone receptor status strongly influences prognosis and therapeutic decision-making [31]. Our findings are broadly consistent with previous studies reporting a predominance of ER and PR positive tumors and a substantial proportion of HER-2 negative cases among women with breast cancer [32–34]. Although the exact proportions vary across studies, such differences may reflect variation in ethnicity, population structure, sample size, tumor characteristics, and methodological approaches to receptor assessment. Overall, the observed hormone receptor profile in our cohort supports the importance of evaluating ER, PR, and HER-2 status in this population and provides a useful context for interpreting the relationships between molecular biomarkers and tumor phenotype.

Another important observation was the significant association between the IGFBP-3 A-202C polymorphism and PR positivity, as well as combined ER/PR positivity. This suggests that the IGFBP-3 polymorphism may be more closely related to hormone receptor biology than to broader clinicopathological features, as no significant associations were observed with ER status alone, HER-2 status, or breast cancer stage. Our findings are partly consistent with those of Ma et al., who also reported no significant association between the IGFBP-3 A-202C genotype and ER or HER-2 status. However, unlike our study, they did not observe a significant association with PR status [33]. This discrepancy may be due to differences in how PR status is defined and measured across studies, including variation in IHC techniques or receptor positivity thresholds. Additionally, the influence of the IGFBP-3 A-202C polymorphism on PR expression may be modulated by the hormonal environment, which can vary with age, menopausal status, and other physiological factors.

We also observed a significant positive association between serum IGF-1 levels and the combined hormone receptor sensitivity groups ER(+)/PR(+) subtype. This finding may indicate that higher circulating IGF-1 is linked to a more hormonally responsive tumor phenotype. The combined double-positive ER(+)/PR(+) receptor status may reflect more accurately the estrogen responsiveness of breast cells and tumor tissue compared with the ER status alone, as the expression of PR is estrogen dependent and hence also depends on the presence of a functional ER, which highlights the interplay between estrogen and progesterone in the development and progression of breast cancer [35]. Our findings align with previous research performed by Kaaks et al. that suggested a link between higher serum IGF-1 levels and hormone receptor-positive breast cancer. In their study, the odds ratios for the highest versus lowest tertiles of serum IGF-1 were 1.66 (95% CI 1.10, 2.50, *p* = 0.004) for ER(+) tumors and 1.77 (95% CI 1.01, 3.10, *p* = 0.007) for ER(+)/PR(+) tumors [36]. In contrast, Papadakis et al. reported no significant correlations between serum IGF-1 levels and combined hormone receptor sensitivity (*p* > 0.05) [32]. These discrepancies may reflect differences in study design, patient populations, and methodology, but they suggest variability in how IGF-1 influences breast cancer characteristics. Despite these differences, our results support the potential interaction between IGF-1 signaling and hormone receptor biology in breast cancer.

Most breast cancer cases in this cohort were diagnosed at Stage II or III, with relatively few patients presenting at Stage IV. This pattern may reflect local clinical presentation in the Gaza Strip, where delayed diagnosis and limited access to specialized oncology services are common. However, it may also have been influenced by the hospital-based recruitment strategy and the inclusion of newly diagnosed, treatment-naive patients. Within this stage distribution, our study found that higher serum IGF-1 levels were significantly associated with more advanced breast cancer stage, particularly Stages III and IV. Although these correlations were modest, they suggest that elevated circulating IGF-1 may be linked to more aggressive disease or greater tumor burden. This interpretation is broadly consistent with the known mitogenic and anti-apoptotic role of IGF-1. However, given the cross-sectional design, this relationship should be interpreted cautiously and considered exploratory. Further validation in larger, longitudinal, and multi-center studies is required before any clinical application can be considered.

Our results are consistent with those of Morgillo et al., who observed a strong correlation between high basal serum IGF-1 levels and advanced tumor stages [37]. By contrast, Raval and Trivedi found an inverse correlation between tumoral IGF-1 expression and breast cancer stage. Their study focused on tumoral IGF-1 expression, whereas our analysis examined serum IGF-1 levels [38]. Similarly, Khaddour et al. reported no correlation between serum IGF-1 levels and tumor stage or lymph node metastasis in Syrian women with breast cancer [39]. This inconsistency could be attributed to several factors, including genetic differences, environmental influences, variations in sample size, study design, population characteristics, and whether IGF-1 levels were measured in serum or tumor tissue.

Serum IGFBP-3 showed no consistent association with clinicopathological features in this study, suggesting a complex, context-dependent role in breast cancer biology. IGFBP-3 may act through both IGF-dependent and IGF-independent mechanisms, with its effects potentially varying by tumor subtype, hormonal environment, and interactions with other signaling pathways. The absence of consistent findings may reflect the biological heterogeneity of breast cancer.

This study has several limitations that should be considered when interpreting the findings. First, the relatively small sample size may limit statistical power, particularly for subgroup analyses. Second, the study was conducted in a single geographic region, the Gaza Strip, which may limit the generalizability of the findings to other populations with different environmental, nutritional, and genetic backgrounds. Third, although the present analysis addresses a distinct research objective, it was conducted using the same cohort as our previous publication, and the novelty of these findings should be interpreted accordingly. Finally, the cross-sectional design precludes any conclusions regarding causality or disease progression. Therefore, the associations identified in this study should be regarded as exploratory and hypothesis-generating. Larger, longitudinal, and multi-center studies are needed to validate these findings and further clarify the role of IGF-1, IGFBP-3, and genetic variation in breast cancer biology.

Despite these limitations, this study provides valuable data from an underrepresented population and offers an integrated analysis of genetic, biochemical, and immunohistochemical factors in breast cancer. In a setting such as the Gaza Strip, where the breast cancer burden is substantial and resources are constrained, understanding the biological relationships among these markers may help guide future research and generate hypotheses for more personalized approaches to breast cancer assessment and management.

## 5. Conclusion

This study provides an integrated assessment of IGFBP-3 A-202C polymorphism, serum IGF-1 and IGFBP-3 levels, and hormone receptor status in Palestinian women with breast cancer from the Gaza Strip. A high prevalence of hormone receptor positivity was observed in this cohort. Moreover, the findings suggest that the IGFBP-3 A-202C genotype may be associated with PR positivity and combined ER/PR positivity, while elevated serum IGF-1 levels were associated with the ER(+)/PR(+) subtype and more advanced disease stage. These results support the potential biological relevance of IGF-axis biomarkers and IGFBP-3 genetic variation in hormone receptor-related breast cancer characteristics. Although the cross-sectional design precludes causal inference, the observed associations suggest that these markers may contribute to a better understanding of tumor heterogeneity and disease behavior in this population. Further validation in larger, longitudinal, and multi-center studies is needed to confirm these findings and to determine whether these biomarkers may have future value in breast cancer stratification and management. By providing evidence from an underserved and underrepresented population, this study contributes to a deeper understanding of the molecular heterogeneity of breast cancer and offers a foundation for future biomarker-driven research in similar settings.

## Supporting information

S1 File Inclusivity in global research questionnaire (1). (DOCX)

S1 Dataset Dataset. (XLS)

## Abbreviations

IGF-1: insulin-like growth factor-1
IGFBP-3: insulin-like growth factor binding protein 3
ER: estrogen receptor
PR: progesterone receptor
HER-2: human epidermal growth factor receptor 2
IHC: immunohistochemistry
SD: standard deviation
CI: confidence interval
OR: odds ratio
